# Phosphorylation of PUF-A/PUM3 on Y259 modulates PUF-A stability and cell proliferation

**DOI:** 10.1371/journal.pone.0256282

**Published:** 2021-08-18

**Authors:** Hung-Wei Lin, Jin-Yu Lee, Nai-Lin Chou, Ting-Wei Shih, Mau-Sun Chang

**Affiliations:** 1 Institute of Biochemical Sciences, National Taiwan University, Taipei, Taiwan; 2 Institute of Biological Chemistry, Academia Sinica, Taipei, Taiwan; University of Louisville, UNITED STATES

## Abstract

Human PUF-A/PUM3 is a RNA and DNA binding protein participating in the nucleolar processing of 7S to 5.8S rRNA. The nucleolar localization of PUF-A redistributes to the nucleoplasm upon the exposure to genotoxic agents in cells. However, little is known regarding the roles of PUF-A in tumor progression. Phosphoprotein database analysis revealed that Y259 phosphorylation of PUF-A is the most prevalent residue modified. Here, we reported the importance of PUF-A’s phosphorylation on Y259 in tumorigenesis. *PUF-A* gene was knocked out by the Crispr/Cas9 method in human cervix epithelial HeLa cells. Loss of PUF-A in HeLa cells resulted in reduced clonogenic and lower transwell invasion capacity. Introduction of PUF-A^Y259F^ to PUF-A deficient HeLa cells was unable to restore colony formation. In addition, the unphosphorylated mutant of PUF-A, PUF-A^Y259F^, attenuated PUF-A protein stability. Our results suggest the important role of Y259 phosphorylation of PUF-A in cell proliferation.

## Introduction

Pumilio/fem-3 (PUF) proteins belong to the members of *Drosophila melanogaster* Pumilio and *Caenorhabditis elegans* fem-3 mRNA binding factors [1,2], which contain of 8–12 conserved α-helical Pumilio (PUM) repeats [3,4]. Each PUM repeat consists of 35 to 39 amino acids capable of associating with the 3’-untranslated region (3’-UTR) of target mRNAs to promote mRNA degradation and translational repression [5–9]. In each PUM repeat, there are three α helices and the second α helix contains the tripartite recognition motif (TRM) that recognizes a specific RNA base [5–9]. Structurally, the interaction of PUF proteins with different RNA elements is mediated by a two-way mechanism, of which one set of PUM repeats recognizes a conserved 5′-UGUA sequence, while the other set of PUM repeats recognizes a variable 3′-element [5–9].

PUF-A (also known as PUM3, Pumilio RNA binding family member 3, an ortholog of yeast Puf6) recognizes structured RNA and participates in pre-ribosomal RNA processing [10,11]. Ribosome biogenesis requires hundreds of factors in the processing of ribosomal RNAs and assembly of rRNAs and ribosomal proteins into the large ribonucleoprotein complex. The pre-rRNA undergoes multiple trimming steps to remove several transcribed spacers and generate the mature rRNAs [12–15]. PUF-A is critical for the 5.8S small ribosomal subunit assembly [10,11,15]. Structurally, the N-terminal region of PUF-A contains three PUM repeats (N-R1 to N-R3, residues 131–277) flanked by an N-terminal pseudo-repeat (N-R1′). By contrast, the C-terminal subdomain has eight PUM repeats (C-R1 to C-R8, residues 278–646) and a C-terminal pseudo-repeat (C-R8′) [11]. Alterations in the expression level of human PUF-A are associated with breast cancer, autoimmunity, and learning impairment [16,17].

Previously, we have shown that PUF-A predominantly localizes in the nucleoli. The nucleolar localization of PUF-A would redistribute to the nucleoplasm after the exposure to RNA polymerase inhibitors [actinomycin D (ActD) and 5,6-dichlorobenzimidazole riboside (DRB)] and topoisomerase inhibitors [camptothecin (CPT) and etoposide]. PUF-A specifically interacts with the catalytic domain of PARP-1 and inhibits poly(ADP-ribosyl)ation of PARP-1 *in vitro* [18]. We also report the function of PUF-A in promoting breast cancer progression. This promoting effect of PUF-A in tumorigenesis might be correlated with the regulation of its associated mRNAs, such as *RbAp48* and *DDX3* [16]. In this study, we addressed the importance of phosphorylation of PUF-A on Y259. Unphosphorylated PUM^Y259F^ mutant affected the protein stability and subcellular localization of PUF-A in response to genotoxic stress. Further studies have revealed that the phosphorylation of PUF-A on Y259 is important for cell proliferation.

## Materials and methods

### Cell lines

Human HeLa and HEK293T cells were obtained from the American Type Culture Collection (ATCC; Rockville, MD) and cultured in DMEM medium (Hyclone, Utah, USA) supplemented with 10% FBS. All cell lines were submitted to real time PCR for mycoplasma detection and short tandem repeats identification by capillary electrophoresis for cell line authentication.

### Antibodies

The mouse anti-PUF-A monoclonal antibody has been described [18]. Mouse anti-HA (Cat. No. SC-7392) was purchased from Santa Cruz Biotech (Santa Cruz, CA). Mouse anti-pTyr antibody was purchased from Santa Cruz Biotech (Cat. No. SC-7020, Santa Cruz, CA). Mouse anti-GAPDH antibody was from Novus (Cat. No. NB-300221, Littleton, CO).

### sgRNA-mediated gene deletion

Two sgRNA oligos were designed to delete exon 2 containing ATG translation start site using the following sequences, sgRNA#1, 5’-AACTTCCATCGTAGCAACTC-3’ and sgRNA#2, 5’-TAACAGCCAAACACCCACAT-3’. Briefly, these two sgRNAs were cloned into an all-in-one sgRNA/Cas9 expression lentivector. HeLa cells were plated at 40–50% confluence and transfected with pU6-sgRNA/Cas9 plasmid using TrnasIT-LT1 reagent (Cat. No. MIR2300, Mirus Bio, Madison, WI) for 96 h. A surrogate vector containing EGFP and mCherry was used to monitor the transfection efficiency. A serial dilution of HeLa cells was conducted to isolate a single cell colony. In total, fifty-five clones were grown and Immunofluorescent/immunoblotting analyses were performed to examine the knockout efficiency. Independent clones of PUF-A-ablated HeLa cells by CRISPR-Cas9 mediated gene deletion were isolated for the following experiments.

### Immunofluorescence

HeLa cells grown on coverslips were fixed in 4% paraformaldehyde in PBS for 20 min and permealized with 0.5% Triton for 5 min. Cells were incubated with anti-PUF-A or anti-HA primary antibodies and rhodamine-conjugated secondary antibodies. Immunostained images were recorded using a Leica upright microscope (Leica, Germany).

### Immunoblotting

Protein extracts were solubilized in RIPA buffer (25 mM Tris-HCl pH 7.5, 150 mM NaCl, 1% NP-40, 1% sodium deoxycholate, 0.1% SDS, 0.1 mM sodium orthovanadate, 10 mM β-glycerophosphate, protease inhibitors) on ice for 30 min and sonicated for 7 min using a Misonix S-4000 sonicator (Farmingdale, NY). The supernatants were collected by centrifugation at 13,500 rpm for 15 min at 4°C. Thirty micrograms of proteins were subjected to SDS-PAGE electrophoresis, transferred to nitrocellulose membranes, and immunoblotted with the indicated antibodies. The membranes were then incubated with enhanced chemiluminescent HRP substrate (Supersignal West Pico, Thermo Scientific) for 3 min and followed by exposure to X-ray film.

### Clonogenic formation assay

HeLa cells were trypsinized and number counted by a hemocytometer. Totally, 1x10^3^ control and *PUF-A* knockout HeLa cells were seeded in DMEM complete growth media and allowed to grow for 12 days until visible colonies formed. Colonies were stained with 0.25% crystal violet in ddH_2_O, washed with PBS twice, and air dried. The stained colonies with diameter larger than 0.1 mm were counted.

### Cell invasion assay

Forty-eight hours post transfection, matrigel invasion assays were conducted using 8 μm Transwell chambers. Matrigel was diluted in distilled water, added to the upper wells of Transwell chambers (2 mg/well), and dried in a sterile hood for 3 h at 37°C before the addition of cells. Cells at a concentration of 1x10^4^ cells with 300 μl of serum-free medium containing 0.1% FBS were seeded into the upper chamber. 700 μl of medium containing 10% FBS was added into the lower wells. After 24 h for invasion assays, cells on the underside of the membrane of chambers were fixed in methanol and then stained with crystal violet. Invaded cells were recorded under a microscope and counted from three independent experiments.

### MTT assay

Cells were seeded in a 24-well tissue culture plate and treated with CPT at the indicated concentrations. After 24 h, MTT (Sigma-Aldrich) solution (0.5 mg/mL) was added to each well. After incubation for 2 h at 37°C, formazan crystals in viable cells were solubilized in 200 mL of DMSO. The soluble formazan product was quantified using an ELISA reader at 570 nm.

### Statistical analysis

Analysis was carried out using GraphPad Prism 6 software. All values were expressed as mean ± SD. The paired Student’s *t*-test (two-tailed) was used to calculate the statistical significance of differences between groups. The *p* < 0.05 was considered statistically significant.

## Results

### PUF-A is associated with overall and progression free survival in cancer patients

**Fig 1:**
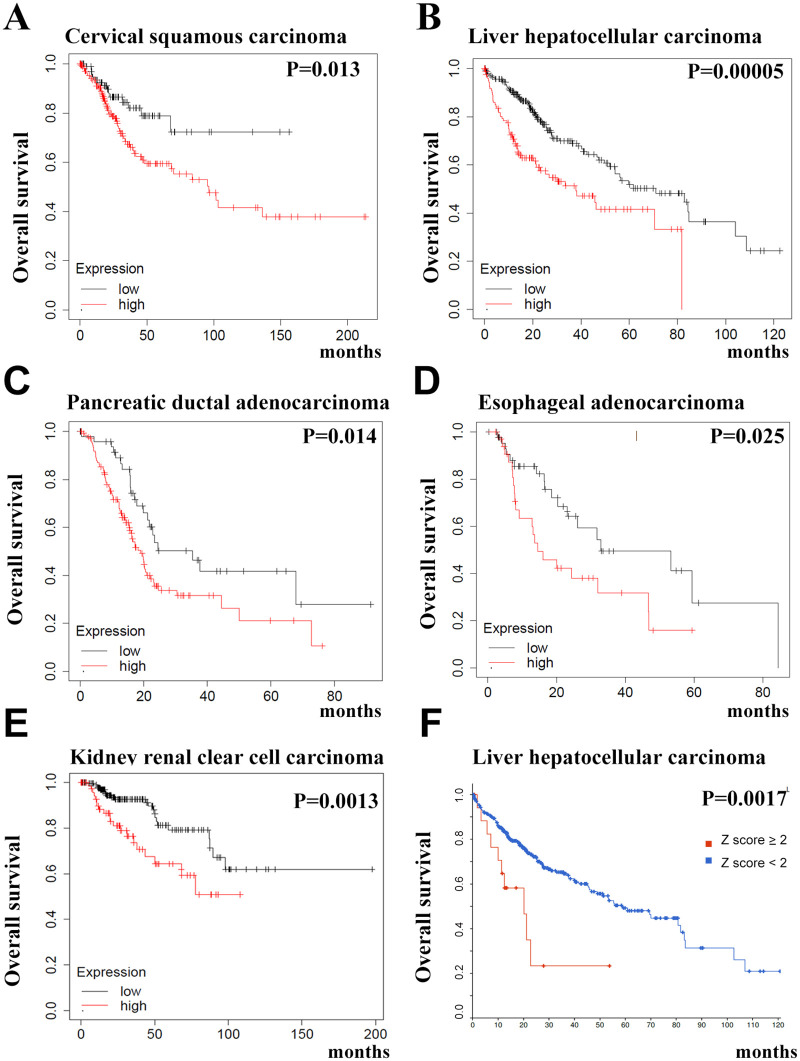
*PUF-A* expression level is associated with the overall survival of cancer patients. (A) Cervical squamous carcinoma. n = 304. (B) Liver hepatocellular carcinoma. n = 371. (C) Pancreatic ductal adenocarcinoma. n = 177. (D) Esophageal adenocarcinoma. n = 80. (E) Kidney renal papillary cell carcinoma. n = 288. The cohorts were divided into two groups, high (red) and low (black), according to the median expression value of *PUF-A*, which were retrieved from the Kaplan–Meier plotter database (http://kmplot.com/analysis/index.php?p=service&cancer). (F) Liver hepatocellular carcinoma. n = 365. Patient data was obtained from cBioPortal TCGA PanCancer Atlas dataset. Z score ≥ 2 (red).

To investigate the roles of PUF-A in cancer progression, we examined the association of the *PUF-A* expression level with overall survival in cancer patients. Using the Kaplan Meier Plotter (http://kmplot.com/analysis/index.php?p=service&cancer) [19], a publicly accessible database, a high level of PUF-A mRNA is noticeably associated with poor survival in cervical squamous carcinoma, liver hepatocellular carcinoma, pancreatic ductal adenocarcinoma, esophageal adenocarcinoma and kidney renal clear cell carcinoma (Fig 1A-1E). Additionally, an elevated *PUF-A* expression is associated with poor survival in liver hepatocellular carcinoma using the cBioportal TCGA dataset analysis (Fig 1F), supporting the notion that high *PUF-A* expression is associated with poor survival in cancer patients.

### PUF-A ablation affected colony formation in HeLa cells

**Fig 2:**
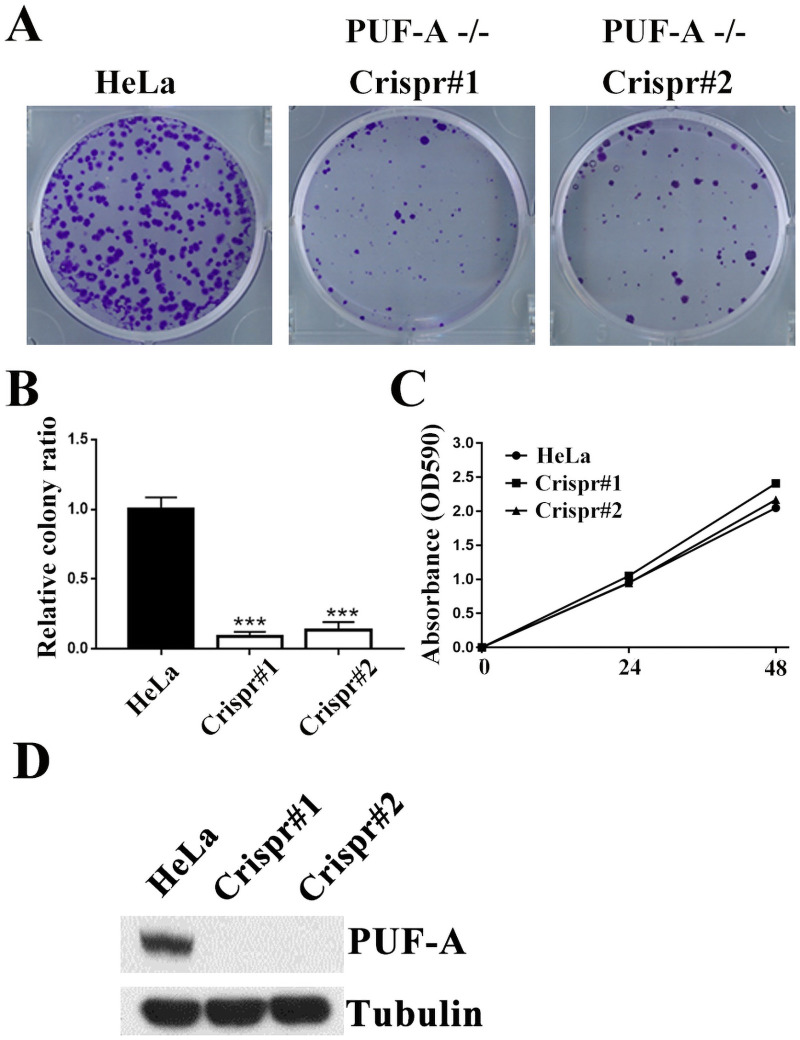
Clonogenic assays in PUF-A-ablated HeLa cells. (A) 800 cells were plated on 6-well dishes and grown for 10–14 days. Cells were stained with crystal violet. Colonies larger than 0.1 mm in diameter were scored. (B) Quantitative results are shown and each bar represents the mean ± SD of three independent experiments. ***P < 0.001. (C) Cell proliferation was determined by MTT assay. (D) Cell extracts isolated from control and PUF-A-depleted HeLa cells were immunoblotted with the indicated antibodies. All Western blots were processed in identical conditions and cropped from S4 Fig.

To illustrate whether PUF-A contributed to clonogenic formation ability, we knocked out PUF-A’s expression in HeLa cells by the Crispr-Cas9 gene editing method, which was conducted by the RNA Technology Platform and Gene Manipulation Core in Taiwan. Compared with control HeLa cells, the number of colonies was significantly reduced in PUF-A-ablated HeLa cells (Fig 2A & 2B). However, control and PUF-A depleted HeLa cells exhibited a similar proliferation rate when cultured on solid matrix (Fig 2C). Immunoblot analysis showed that the expression level of PUF-A was completely depleted in PUF-A-ablated HeLa cells (Fig 2D). The results indicate that PUF-A is required for colony formation in HeLa cells.

### PUF-A affected transwell invasion

**Fig 3:**
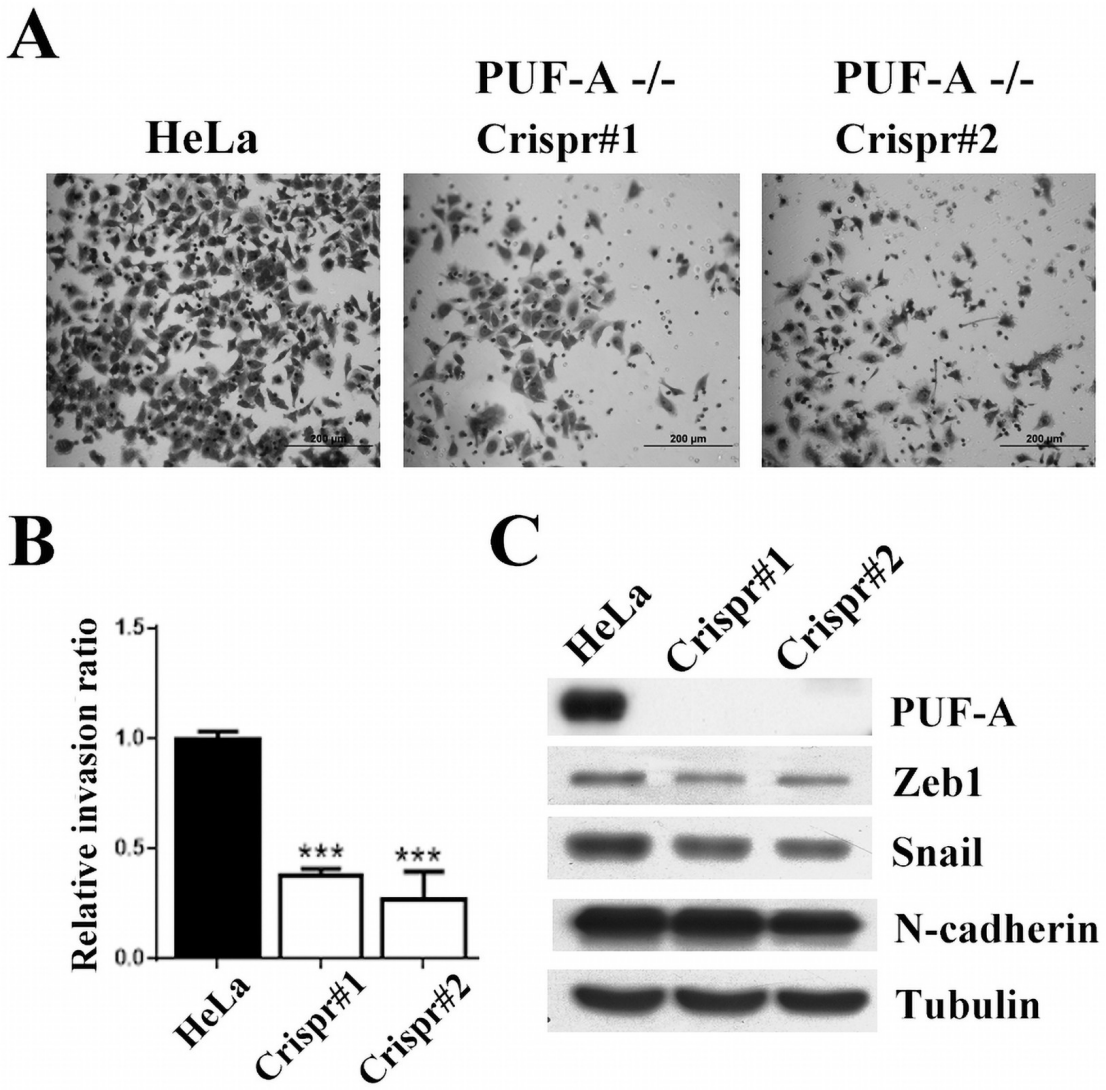
PUF-A promotes cell invasion *in vitro*. (A) PUF-A-depleted HeLa cells were loaded onto the Matrigel for invasion assay. Cells moving to the lower surface of the membrane were stained with crystal violet and photographed. Invasion ratio was determined by the number of migrated cells in a confined area. Scale bar, 0.2 mm. (B) Invasion ratio was quantified. Each bar represents the mean ± SD of three independent experiments. **P < 0.001. (C) Cell extracts isolated from control and PUF-A-depleted HeLa cells were immunoblotted with the indicated antibodies. All Western blots were processed in identical conditions and cropped from S4 Fig.

To examine whether PUF-A potentiates cell invasion, a transwell apparatus coating with Matrigel to mimic the microenvironment of invasion was conducted. The majority of control HeLa cells penetrated the Matrigel within 24 h; nonetheless, most of the PUF-A-depleted cells were unable to travel to the lower chamber (Fig 3A). Quantitative results showed that the absence of PUF-A reduced transwell invasion at least by 50% (Fig 3B). Since epithelial-mesenchymal transition (EMT) is considered as an important event for cell migration and invasion, the expression levels of EMT markers, such as N-cadherin, and EMT transcription factors Snail and Zeb1, were examined in PUF-A-depleted HeLa cells. E-cadherin is not expressed in the HeLa cells. As anticipated, decreased protein expression of Zeb1, N-cadherin, and Snail were found in PUF-A-depleted cells (Fig 3C), indicating that PUF-A might modulate the expression of the EMT’s markers and thus cell migration and invasion.

### Phosphorylation of PUF-A determined its subcellular localization

**Fig 4:**
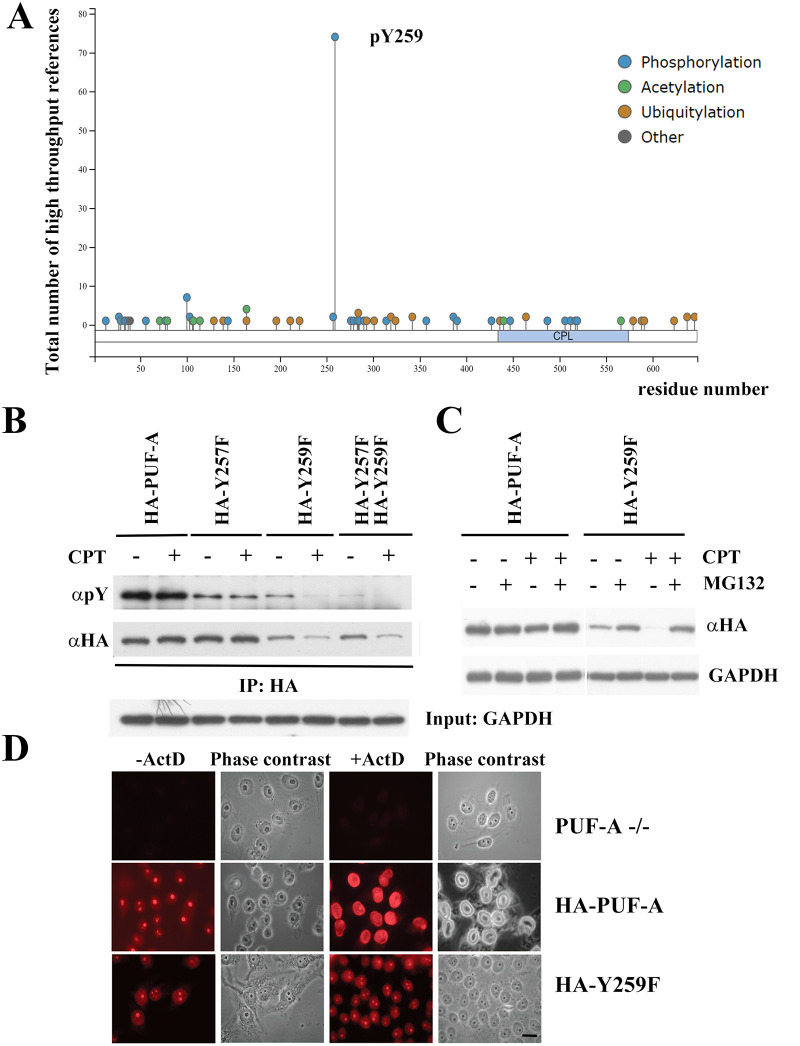
Phosphorylation of PUF-A on Y259. (A) Identification of phosphorylated PUF-A at Y259 using PosphoSitePlus database. (B) HA-PUF-A, HA-PUF-A^Y257F^ (HA-Y257F), HA-PUF-A^Y259F^ (HA-Y259F) and HA-PUF-A^Y257FY259F^ (HA-Y257F/HA-Y259F) transfected HEK293T cells were incubated with camptothecin (CPT, 5 μM) for 3 hr. Cell extracts were immunoprecipitated using anti-HA agarose and then immunoblotted with indicated antibodies. All Western blots were processed in identical conditions and cropped from S4 Fig. (C) HA-PUF-A and HA-PUF-A^Y259F^ transfected HEK293T cells were incubated with MG132 for 3 h prior to CPT treatment for 16 h. Immunoblotting was carried out with indicated antibodies. All Western blots were processed in identical conditions and cropped from S4 Fig. (D) HA-PUF-A and HA-PUF-A^Y259F^ were transfected into PUF-A ablated HeLa cells. Cells were exposed to actinomycin D (ActD, 1 μg/μl) for 1 h and then immunofluorescence staining was conducted. Scale bar, 10 μm.

Since PUF-A interacts with PARP1 and both redistribute from the nucleolus to the nucleoplasm upon genotoxic stress [18], we wondered whether poly(ADP-ribosyl)ation or other post translational modifications could change the subcellular localization of PUF-A. However, no poly(ADP-ribosyl)ation was found in PUF-A after camptothecin (CPT, an inhibitor of topoisomerase I) exposure. Next, we addressed whether phosphorylation had any impact on the distribution of PUF-A. HeLa cells were exposed to calyculin A, a serine/threonine phosphatase inhibitor, or sodium orthovanadate, a tyrosine phosphatase inhibitor, for 1 h prior to the CPT treatment. Immunofluorescent results showed that the relocalization of PUF-A to the nucleoplasm upon CPT treatment was inhibited in the presence of calyculin A or sodium orthovanadate (S1A Fig), indicating that protein dephosphorylation was important for PUF-A’s translocation to the nucleoplasm. Meanwhile, HA-PUF-A transfected HeLa cells were exposed to 5,6-dichlorobenzimidazole riboside (DRB, an inhibitor of RNA polymerase II) or CPT for subsequent immunoprecipitation and immunoblot analyses. The results showed that the serine and threonine phosphorylation of PUF-A was little changed in response to DRB and CPT treatments. By contrast, the overall tyrosine phosphorylation of PUF-A was significantly decreased (S1B Fig) as compared with those of control cells, indicating that PUF-A is tyrosine phosphorylated in the nucleolus but undergoes tyrosine dephosphorylation upon genotoxic stress. We found that the Y259 residue of PUF-A was likely phosphorylated, which will be described in detail in Fig 4A. To illustrate whether tyrosine phosphorylation was required for the subcellular localization of PUF-A, HA-PUF-A, HA-PUF-A^Y257F^ and HA-PUF-A^Y259F^ were expressed in HeLa cells. Unlike HA-PUF-A and HA-PUF-A^Y257F^, HA-PUF-A^Y259F^ remained in the nucleolus post CPT treatment (S1C Fig), indicating that the tyrosine dephosphorylation of PUF-A on Y259 was essential for PUF-A’s nucleoplasm relocalization in response to genotoxic stress.

### Phosphorylation of PUF-A on Y259 is important for PUF-A stability

To gain more information regarding the biological significance of tyrosine phosphorylation on PUF-A, we accessed the PhosphoSitePlus at https://www.phosphosite.org//homeAction.action to analyze potential post-translation modifications (PTM) sites of PUF-A. Interestingly, the phosphorylation of PUF-A on Y259 displayed the highest score in many high-throughput references (Fig 4A), indicating that pY259 is likely a phosphorylation residue on PUF-A. To elucidate the correlation of pY259 at PUF-A with cell proliferation, we substituted tyrosine 259 with phenylalanine in PUF-A (hereafter PUF-A^Y259F^). We also mutated tyrosine 257 with phenylalanine (PUF-A^Y257F^) as a control and then expressed these mutants in HEK293T cells. Immunoprecipitation and immunoblotting analyses were carried out to determine the expression levels of PUF-A^Y259F^ and PUF-A^Y257F^ in response to CPT exposure. The expression levels of HA-PUF-A and HA-PUF-A^Y257F^ were similar in respective expressing cells. Intriguingly, the protein levels of HA-PUF-A^Y259F^ and HA-PUF-A^Y257F/Y259F^ were much lower than those of HA-PUF-A and HA-PUF-A^Y257F^ expressing cells in the presence of CPT (Fig 4B), suggesting that HA-PUF-A^Y259F^ protein was unstable upon CPT exposure.

To verify this speculation, HA-ubiquitin was transfected into HEK293T cells and cells were exposed to CPT for 18 h in the presence or absence of MG132, a proteasome inhibitor. Remarkably, the amount of endogenous HA-polyubiquitinated PUF-A was increased in the presence of MG132 (S2 Fig), indicating that the protein level of PUF-A was regulated by the ubiquitin-mediated proteasome degradation. In addition, HA-PUF-A and HA-PUF-A^Y257F^ were quite stable after 3 h CPT treatment while HA-PUF-A^Y259F^ was prone to degradation (Fig 4B). Again, the protein level of PUF-A^Y259F^ was restored in the presence of MG132 after CPT treatment (Fig 4C), echoing the speculation that PUF-A^Y259F^ was degraded by the ubiquitin-proteasome system upon genotoxic challenge. To examine the biological properties of the PUF-A^Y259F^ mutant, HA-PUF-A and HA-PUF-A^Y259F^ were expressed in PUF-A-deficient (PUF-A^-/-^) HeLa cells for immunofluorescence analysis. There was no nucleolar signal in PUF-A ablated cells. By contrast, HA-PUF-A transfection restored PUF-A’s localization in the nucleolus and relocated to the nucleoplasm post actinomycin D (ActD) treatment (the middle panel, Fig 4D). Noticeably, similar to S1C Fig, PUF-A^Y259F^ was still confined in the nucleolus post ActD treatment (the lower panel, Fig 4D), indicating that dephosphorylated Y259 was important for PUF-A relocalization to the nucleoplasm.

### PUF-A^Y259F^ does not affect the poly(ADP-ribosyl)ation of PARP-1

It has been shown that approximate 40% of PARP1 proteins localize to nucleoli and translocate to the nucleoplasm upon DNA damage [20,21]. PUF-A is able to interact with PARP1 and modulates its poly(ADP-ribosyl)ation (PAR) activity upon genotoxic stress [18]. We wondered whether PUF-A Y259 phosphorylation affected PARP1 binding and enzyme activity. Interestingly, the PUF-A^Y259F^ mutant increased its association with PARP-1 (S3A Fig). DNA alkylating agent N-methyl-N’-nitro-N’-nitrosoguanidine (MNNG) causes excess DNA breaks and activates PARP1’s PAR activity. The HA-PUF-A^Y259F^ mutant did not compromise the poly(ADP-ribosyl)ation of PARP-1 after MNNG treatment as compared with HA-PUF-A (S3B Fig). Furthermore, the PUF-A^Y259F^ mutant did not affect cell death post MNNG treatment in PUF-A ablated HEK293T cells and the PUF-A^Y259F^ mutant did not decrease apoptosis post etoposide treatment in PUF-A ablated U2OS cells. Collectively, the Y259 phosphorylation of PUF-A might not affect PARP1’s response to genotoxic stress.

### PUF-A^Y259F^ reduced the clonogenic formation but not invasion capacity

**Fig 5:**
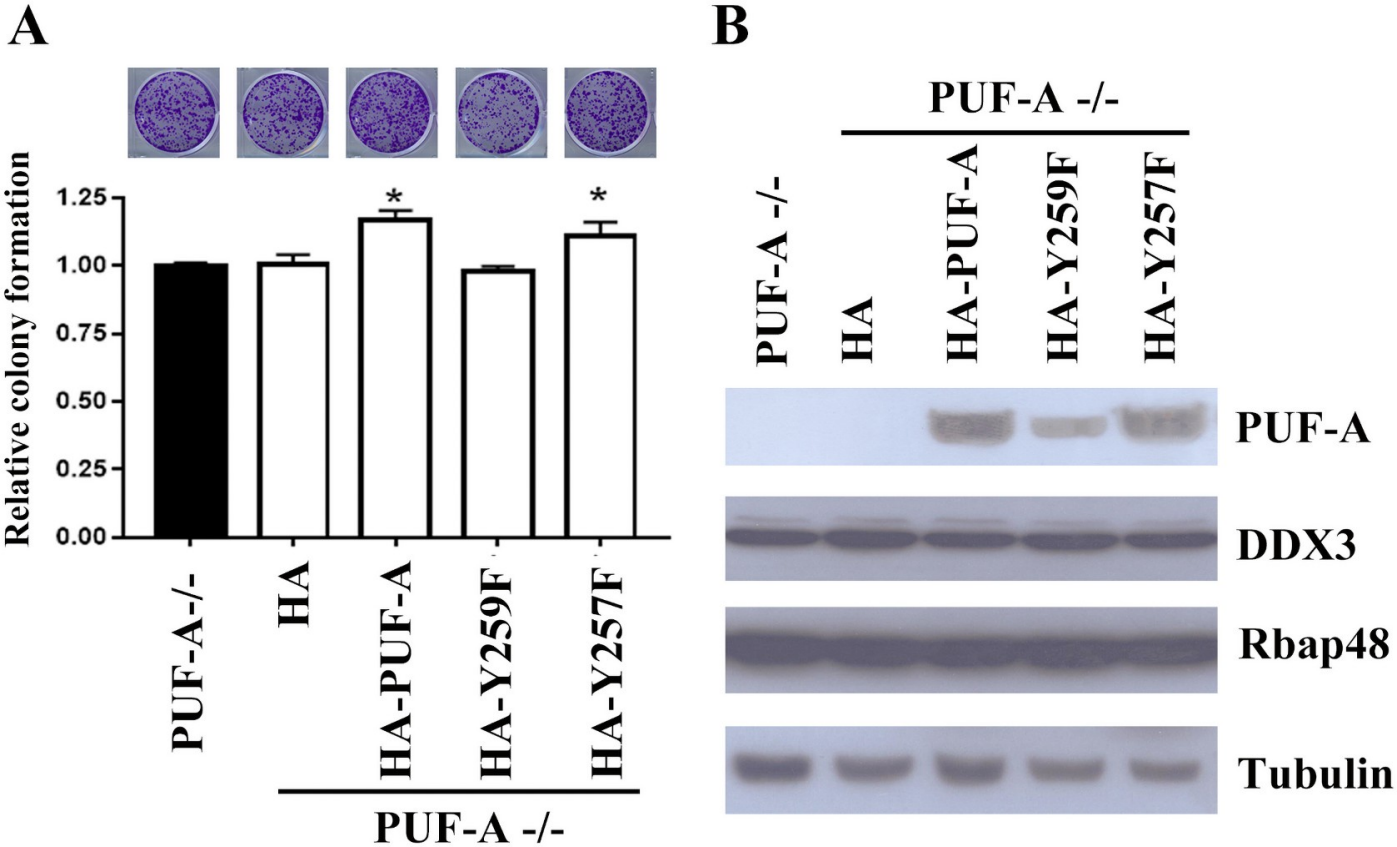
Phosphorylation of PUF-A on Y259 was essential for cell proliferation. (A) HA-PUF-A, HA-PUF-A^Y259F^ (HA-Y259F) and HA-PUF-A^Y257F^ (HA-Y257F) were transfected into PUF-A ablated HeLa cells. 2x10^3^ cells were plated on 6-well dishes and grown for 12 days. Cells were stained with crystal violet and representative images are shown above. Colonies larger than 0.1 mm in diameter were scored. Quantitative results are shown and each bar represents the mean ± SD of three independent experiments. *P < 0.05. (A) Cell extracts were immunoblotted with the indicated antibodies.

To examine whether the Y259 phosphorylation of PUF-A affected cell proliferation, HA-PUF-A, HA-PUF-A^Y259F^ and HA-PUF-A^Y257F^ were transfected into PUF-A-deficient HeLa cells and then colony formation assay was carried out. Compared with PUF-A-deficient cells, HA-PUF-A and PUF-A^Y257F^ increased the number of colonies in PUF-A-deficient cells. By contrast, PUF-A^Y259F^ was unable to restore the clonogenic capacity in PUF-A-deficient HeLa cells (Fig 5A), indicating that Y259 phosphorylation was required for cell proliferation. Immunoblot analysis confirmed that HA-PUF-A^Y259F^ and HA-PUF-A^Y257F^ were expressed in HeLa cells as well DDX3 and RbAp48 (Fig 5B), which are two PUF-A associated RNAs [16]. Additionally, the expression of PUF-A^Y259F^ did not affect the transwell invasion of HeLa cells.

## Discussion

In summary of our studies, PUF-A expression is associated with overall survival of cancer patients. PUF-A ablation reduces cell proliferation and transwell invasion in HeLa cells. The phosphorylation of PUF-A on Y259 residue is essential for PUF-A’s protein stability and clonogenic formation of HeLa cells. PUF-A^Y259F^ mutant does not prevent PUF-A’s entry into the nucleolus, but affects its leaving from the nucleolus upon genotoxic stress. However, phosphorylation of PUF-A on Y259 neither affects its association with PARP1 nor alters the poly(ADP-ribosyl)ation activity of PARP1.

Induction of DNA damage results in the rapid recruitment of PARP1 to DNA damage sites through its DNA-binding domain. This stimulates the catalytic activity of PARP1 to conduct the synthesis of polyADP-ribose (PAR) chains on itself, surrounding histones and non-histone proteins [22]. DNA damage proteins that interact non-covalently with PAR generally contain PAR-binding motifs (PBMs), PAR-binding zinc finger motifs (PBZs), macrodomains, WWE domains and other modules [23]. Although there is no canonical PBM in PUF-A, PUF-A interacts with the catalytic domain of PARP1 and inhibits its PAR activity *in vitro* [18]. In S4 Fig, PUF-A^Y259F^ mutant pulled down more PARP1 protein compared with wild-type PUF-A. However, this increased association did not further compromise PARP1’s PAR enzymatic activity, indicating that PUF-A^Y259F^ mutant may provide a physical interaction with PARP1 in the nucleolus instead of reducing PARP1 activity. Interestingly, the functions of PARP1 in the nucleolus not only modulate the rRNA transcription [24] but also facilitate the shuttling of DNA damage related proteins, such as WRN and XRCC1, from the nucleolus to the nucleoplasm [25]. With regard to DNA damage, if PUF-A^Y259F^ protein remained in the nucleolus under genotoxic stress, more PARP1 could be entrapped in the nucleolus and this may result in the reduction of nucleolar DNA damage proteins redistributed to the nucleoplasm and subsequently impair downstream PARP1-mediated DNA damage repair, which results in an unfavorable condition for cells to survive under DNA genotoxic stress.

The eukaryotic ribosome consists of two subunits formed by the intricate association of ribosomal proteins with four distinct ribosomal RNAs (rRNAs). The small subunit (40S) comprises the 18S rRNA assembled to 33 ribosomal proteins, whereas the large subunit (60S) contains the 5S, 5.8S, and 28S rRNAs associated with 46 ribosomal proteins [26]. PUF-A has been shown to associate with 5.8S rRNA within pre-rRNA in the nucleolus of lung cancer H1299 cells and colon cancer HCT116 cells. However, silencing of PUF-A did not alter the steady state levels of mature 5.8S rRNA [26], suggesting that PUF-A may not participate in the processing for the pre-5.8S rRNA. Instead, PUF-A facilitates the assembly and nuclear export of pre-ribosomes [27]. Additionally, silencing of PUF-A decreased the expression of S6 and L5 in the cytoplasm with an element in the nucleolus compared to control cells [27], indicating that PUF-A depletion in HeLa cells might result in the defective assembly and nuclear export of pre-ribosomes.

PUF-A contains eleven PUM repeats to form an L-shaped structure [11], which the L-turn is located between N-R3 and C-R1 repeat. Interestingly, Y259 residue of PUF-A is embedded within the N-R3 PUM repeat and near the C-R1 PUM repeat. This region is probably an important interface for the non-specific association with single- or double-stranded RNA or DNA [11]. Evolutionally, Y259 residue is conserved in homologs of human, mouse, and zebrafish, but not in yeast Puf-6 [18], indicating that Y259 phosphorylation might be a conserved event occurred in vertebrates. Since the dephosphorylated PUM^Y259F^ mutant was targeted to proteasomal degradation, it is most likely that dephosphorylated PUF-A mutant may alter its L-shaped structure and is destined to be polyubiquitinated for degradation.

Phosphorylation provides a dynamic mechanism to regulate protein activity, stability and subcellular localization, which is balanced through the reciprocal phosphorylation/dephosphorylation of kinases and phosphatases [28]. For example, nucleophosmin (NPM) is a phosphoprotein and mainly localized in the nucleolus. NPM is phosphorylated by various kinases at multiple sites and shuttled between the nucleus and the cytoplasm [29]. By contrast, phosphatase PPM1D increases the phosphorylation of NPM via a PPM1D-CDC25C-CDK1-PLK1 signaling pathway to maintain nucleolar formation [30]. We managed to identify potential nucleolar tyrosine kinases and phosphatases which could regulate the phosphorylation status of PUF-A. Using proteomic analysis, tyrosine phosphatase CDC14 and serine/threonine phosphatase PP1 were isolated by this approach. However, we do not have substantial evidence to conclude that CDC14 and PP1, or other kinases, are involved in the phosphorylation of Y259 to maintain the protein stability and nucleolar localization of PUF-A. Additionally, we cannot exclude the possibility that some of PUM^Y259F^ mutant was able to export from the nucleolus to the nucleoplasm. Since PUM^Y259F^ protein is unstable, it may be quickly degraded by the proteasomes in the nucleoplasm upon genotoxic exposure.

PUF proteins mainly participate in stem cell maintenance, organelle biogenesis, oogenesis, neuron function, and memory formation [1]. We have shown the association of PUF-A with RbAp48/RBBP4 and DDX3 in breast cancer MDA-MB-231 cells [16]. Both functions of DDX3 and RbAp48 could contribute to promote tumor progression. Nonetheless, the expression levels of DDX3 and RbAp48 were not changed in PUF-A-ablated HeLa cells, suggesting that DDX3 and RbAp48 were not the main regulatory targets by PUF-A in HeLa cells in promoting tumor formation.

In conclusion, our results imply that the tyrosine phosphorylation of PUF-A on Y259 residue is important for PUF-A stability and cell proliferation. Although we were unable to generate a specific antibody against phospho-Y259 of PUF-A, the phosphorylation of PUF-A on Y259 may become a useful marker in tumor progression.

## Supporting information

S1 Fig Reduced tyrosine phosphorylation of PUF-A post genotoxic treatment. (A) HeLa cells were exposed to camptothecin (CPT, 5 μM) for 1 h after the treatment of calycurin A (10 nM) or sodium orthovanadate (2 mM) for 1 h and then immunostained with anti-PUF-A mAb. Nucleoli were shown in dark spots by phase-contrast images. Scale bar, 10 μm. (B) HeLa cells were exposed to 5,6-dichlorobenzimidazole riboside (DRB, 1 μg/μl) and CPT (5 μM) and whole-cell extracts were collected at the indicated times for Western blot analysis. Band intensity of phosphoproteins was measured by ImageJ software and normalized with internal PUF-A. All Western blots were processed in identical conditions and cropped from S4 Fig. (C) HeLa cells were transfected with HA-PUF-A, HA-PUF-A^Y257F^ or HA-PUF-A^Y259F^ constructs and exposed to camptothecin for 1 h. Cells were immunostained with anti-HA antibody. Bar, 10 μm.
(DOCX)

S2 Fig PUF-A was degraded by polyubiquitination. HA-Ub was transfected into HEK293T cells exposed to CPT (5 μM) for 18 h with or without the addition of MG132 (5 μM) for 6h. Total cell extracts were immunoprecipitated with anti-PUF-A monoclonal antibody and immunoblotted with anti-HA antibody. All Western blots were processed in identical conditions and cropped from S4 Fig.
(DOCX)

S3 Fig PUF-A^Y259F^ did not affect poly(ADP-ribosyl)ation of PARP1. (A) HA-PUF-A, HA- PUF-A^Y259F^, and HA- PUF-A^Y257F/Y259F^ were transfected into HEK293T cells and exposed to CPT (5 μM) for 3 h. Cell extracts were immunoprecipitated by anti-HA beads and then immunoblotted by anti-HA and anti-PARP1 antibodies. All Western blots were processed in identical conditions and cropped from S4 Fig. (B) Empty HA-vector, HA-PUF-A and HA-PUF-A^Y259F^ were transfected into PUF-A deficient HEK293T cells for 48 h and exposed to MNNG (5 μM) for indicated times. Cell extracts were immunoprecipitated by anti-PARP1 antibody and immunoblotted by anti-polyADP-ribose (PAR) antibody. All Western blots were processed in identical conditions and cropped from S4 Fig. (C) Control HA-vector, HA-PUF-A and HA-PUF-A^Y259F^ were transfected into PUF-A ablated HEK293T cells for 48 h and exposed to MNNG (2.5 μM) for 18 h and U2OS cells exposed to Etoposide (50 μM) for 18 h. Apoptotic cells were labeled with FITC-conjugated Annexin V for flow cytometry analysis. No significant difference in response to MNNG and etoposide was found.
(DOCX)

S4 Fig Uncropped images for all gels and Western blots. (DOCX)

S1 File (DOCX)
