# Supplementary material for: Phosphorylation of PUF-A/PUM3 on Y259 modulates PUF-A stability and cell proliferation
Source: PLoS One. 2021 Aug 18;16(8):e0256282. doi: 10.1371/journal.pone.0256282 (PMC8372891; doi:10.1371/journal.pone.0256282)
Supplement: S1 Fig — (A) HeLa cells were exposed to camptothecin (CPT, 5 μM) for 1 h after the treatment of calycurin A (10 nM) or sodium orthovanadate (2 mM) for 1 h and then immunostained with anti-PUF-A mAb. Nucleoli were shown in dark spots by phase-contrast images. Scale bar, 10 μm. (B) HeLa cells were exposed to 5,6-dichlorobenzimidazole riboside (DRB, 1 μg/μl) and CPT (5 μM) and whole-cell extracts were collected at the indicated times for Western blot analysis. Band intensity of phosphoproteins was measured by ImageJ software and normalized with internal PUF-A. All Western blots were processed in identical conditions and cropped from S4 Fig. (C) HeLa cells were transfected with HA-PUF-A, HA-PUF-AY257F or HA-PUF-AY259F constructs and exposed to camptothecin for 1 h. Cells were immunostained with anti-HA antibody. Bar, 10 μm. (DOCX) [file pone.0256282.s001.docx]

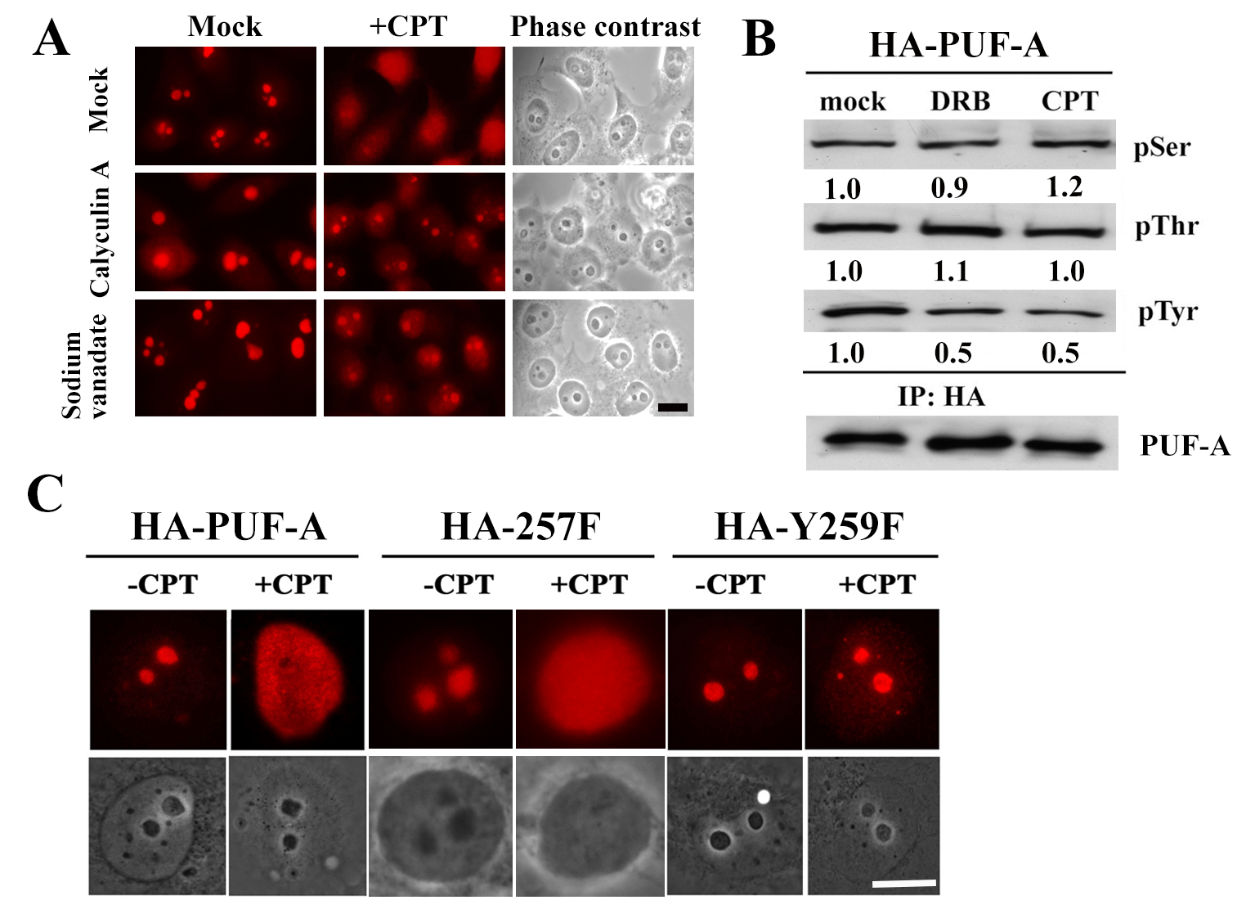


**S1 Fig.** Reduced tyrosine phosphorylation of PUF-A post genotoxic treatment. (A) HeLa cells were exposed to camptothecin (CPT, 5 μM) for 1 h after the treatment of calycurin A (10 nM) or sodium orthovanadate (2 mM) for 1 h and then immunostained with anti-PUF-A mAb. Nucleoli were shown in dark spots by phase-contrast images. Scale bar, 10 μm. (B) HeLa cells were exposed to 5,6-dichlorobenzimidazole riboside (DRB, 1 μg/μl) and CPT (5 μM) and whole-cell extracts were collected at the indicated times for Western blot analysis. Band intensity of phosphoproteins was measured by ImageJ software and normalized with internal PUF-A. All Western blots were processed in identical conditions and cropped from S4 Fig. (C) HeLa cells were transfected with HA-PUF-A, HA-PUF-A^Y257F^ or HA-PUF-A^Y259F^ constructs and exposed to camptothecin for 1 h. Cells were immunostained with anti-HA antibody. Bar, 10 μm.
