# Supplementary material for: Phosphorylation of PUF-A/PUM3 on Y259 modulates PUF-A stability and cell proliferation
Source: PLoS One. 2021 Aug 18;16(8):e0256282. doi: 10.1371/journal.pone.0256282 (PMC8372891; doi:10.1371/journal.pone.0256282)
Supplement: S2 Fig — HA-Ub was transfected into HEK293T cells exposed to CPT (5 μM) for 18 h with or without the addition of MG132 (5 μM) for 6h. Total cell extracts were immunoprecipitated with anti-PUF-A monoclonal antibody and immunoblotted with anti-HA antibody. All Western blots were processed in identical conditions and cropped from S4 Fig. (DOCX) [file pone.0256282.s002.docx]

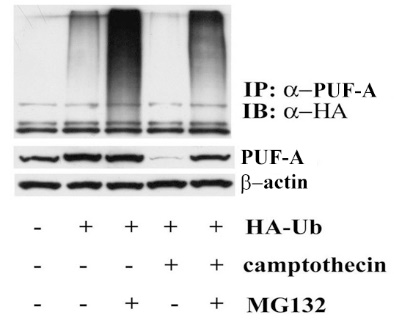


**S2 Fig.** PUF-A was degraded by polyubiquitination. HA-Ub was transfected into HEK293T cells exposed to CPT (5 μM) for 18 h with or without the addition of MG132 (5 μM) for 6h. Total cell extracts were immunoprecipitated with anti-PUF-A monoclonal antibody and immunoblotted with anti-HA antibody. All Western blots were processed in identical conditions and cropped from S4 Fig.
