# Supplementary material for: Phosphorylation of PUF-A/PUM3 on Y259 modulates PUF-A stability and cell proliferation
Source: PLoS One. 2021 Aug 18;16(8):e0256282. doi: 10.1371/journal.pone.0256282 (PMC8372891; doi:10.1371/journal.pone.0256282)
Supplement: S3 Fig — (A) HA-PUF-A, HA- PUF-AY259F, and HA- PUF-AY257F/Y259F were transfected into HEK293T cells and exposed to CPT (5 μM) for 3 h. Cell extracts were immunoprecipitated by anti-HA beads and then immunoblotted by anti-HA and anti-PARP1 antibodies. All Western blots were processed in identical conditions and cropped from S4 Fig. (B) Empty HA-vector, HA-PUF-A and HA-PUF-AY259F were transfected into PUF-A deficient HEK293T cells for 48 h and exposed to MNNG (5 μM) for indicated times. Cell extracts were immunoprecipitated by anti-PARP1 antibody and immunoblotted by anti-polyADP-ribose (PAR) antibody. All Western blots were processed in identical conditions and cropped from S4 Fig. (C) Control HA-vector, HA-PUF-A and HA-PUF-AY259F were transfected into PUF-A ablated HEK293T cells for 48 h and exposed to MNNG (2.5 μM) for 18 h and U2OS cells exposed to Etoposide (50 μM) for 18 h. Apoptotic cells were labeled with FITC-conjugated Annexin V for flow cytometry analysis. No significant difference in response to MNNG and etoposide was found. (DOCX) [file pone.0256282.s003.docx]

**S3 Fig. PUF-A^Y259F^ did not affect poly(ADP-ribosyl)ation of PARP1. (A)** HA-PUF-A, HA- PUF-A^Y259F^, and HA- PUF-A^Y257F/Y259F^ were transfected into HEK293T cells and exposed to CPT (5 μM) for 3 h. Cell extracts were immunoprecipitated by anti-HA beads and then immunoblotted by anti-HA and anti-PARP1 antibodies. All Western blots were processed in identical conditions and cropped from S4 Fig. (**B)** Empty HA-vector, HA-PUF-A and HA-PUF-A^Y259F^ were transfected into PUF-A deficient HEK293T cells for 48 h and exposed to MNNG (5 μM) for indicated times. Cell extracts were immunoprecipitated by anti-PARP1 antibody and immunoblotted by anti-polyADP-ribose (PAR) antibody. All Western blots were processed in identical conditions and cropped from S4 Fig. (**C)** Control HA-vector, HA-PUF-A and HA-PUF-A^Y259F^ were transfected into PUF-A ablated HEK293T cells for 48 h and exposed to MNNG (2.5 μM) for 18 h and U2OS cells exposed to Etoposide (50 μM) for 18 h. Apoptotic cells were labeled with FITC-conjugated Annexin V for flow cytometry analysis. No significant difference in response to MNNG and etoposide was found.
