# Supplementary figures and images for: Phosphorylation of PUF-A/PUM3 on Y259 modulates PUF-A stability and cell proliferation
Source: PLoS One. 2021 Aug 18;16(8):e0256282. doi: 10.1371/journal.pone.0256282 (PMC8372891; doi:10.1371/journal.pone.0256282)

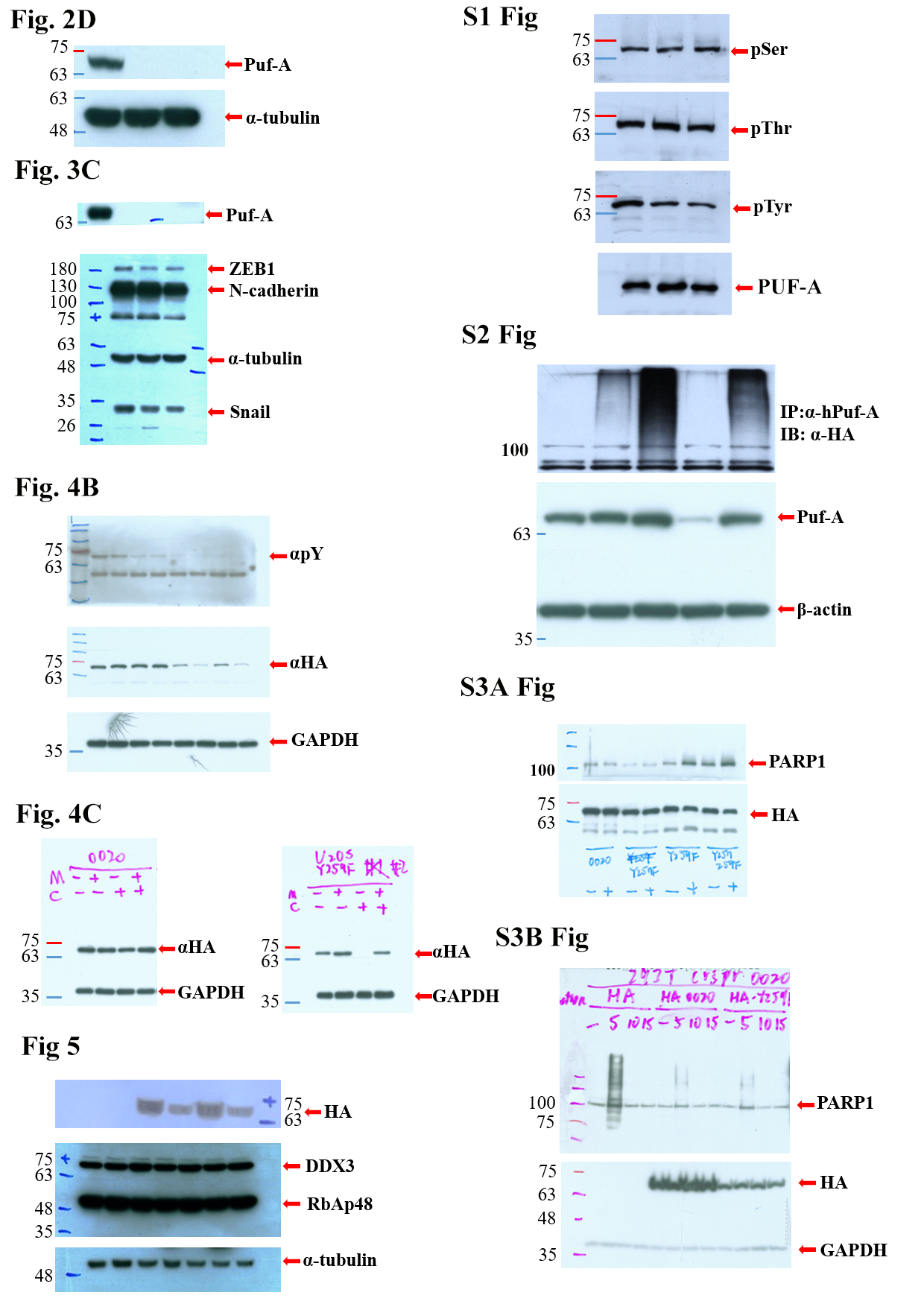

**S4 Fig. Uncropped images for all gels and Western blots.**

Supplement: S4 Fig — (DOCX) [file pone.0256282.s004.docx]
