# Supplementary material for: Phosphorylation of PUF-A/PUM3 on Y259 modulates PUF-A stability and cell proliferation
Source: PLoS One. 2021 Aug 18;16(8):e0256282. doi: 10.1371/journal.pone.0256282 (PMC8372891; doi:10.1371/journal.pone.0256282)
Supplement: S1 File — (DOCX) [file pone.0256282.s005.docx]

**Supplementary Materials and Methods**

**Antibodies.** Mouse anti-poly(ADP-ribose) polymer antibody was from Abcam (Cat. No. ab14459, Cambridge, UK). Mouse anti-pTyr antibody was purchased from Santa Cruz Biotech (Cat. No. SC-7020, Santa Cruz, CA). Rabbit anti-phosphoserine and anti-phosphothreonine antibodies from Merck Millipore (Cat. No. AB1603 and AB1607, Darmstadt, Germany). Mouse anti-β-actin antibody was from Novus Biologicals (Cat. No. NB-600501, Littleton, CO).

***In vivo* ubiquitination**

HEK293T cells were transfected with HA-tagged ubiquitin (Ub) with the addition of MG132 (5 μM, Sigma-Aldrich) for 6 h or in the presence of CPT (5 μM, Sigma-Aldrich) for 18 h . Total cell extracts were harvested with RIPA buffer and immunoprecipitated by anti-PUF-A antibody for immunoblotting analysis.

**Detection of apoptotic cells**

Apoptotic cells were labeled with FITC-conjugated Annexin V detection kit (Cat. No. 556547, BD, Biosciences, San Jose, CA) for 15 minutes in the dark and analyzed by FACSCalibur (Becton Dickinson).
